# Antibacterial Activity of Various Plants Extracts Against Antibiotic-resistant* Aeromonas hydrophila*

**DOI:** 10.5812/jjm.11370

**Published:** 2014-07-01

**Authors:** Shaza Anwar Al Laham, Frdoos Mohammad Al Fadel

**Affiliations:** 1 Department of Pharmacology and Toxicology, Faculty of Pharmacy, Damascus University, Damascus, Syria

**Keywords:** Antibacterial Properties, Disk Diffusion Test, Plants, *Aeromonas hydrophila*

## Abstract

**Background:**

*Aeromonas hydrophila* cause one of the most important diseases in fishes and lead to economic losses, and may be contaminated human beings.

**Objectives:**

The current research aimed to investigate the anti-bacterial activity shown by the extracts prepared from different parts of *Olea europea*, *Myrtus communis*, *Thymus vulgaris*, *Rosmarinuis officinalis*, and *Achillea falcata* that grow in Syria against *A. hydrophila* that causes the most dangerous bacterial diseases in fish.

**Materials and Methods:**

The study was performed in four stages: First of all, the presence of *A. hydrophila* was investigated in 450 Samples of *Cyprinus Carpio* fish using blood agar, Trypticase soya agar, and Analytical Profile Index (API20E). Secondly, the plants extract was obtained using water, absolute alcohol, then ether using Soxhlet extraction apparatus and rotary vacuum evaporator. Thirdly, the antibacterial activity of some antibiotics on these bacteria was evaluated by disk diffusion method. Finally, the antibacterial effect of the extracts was determined by disk diffusion method.

**Results:**

The studied antibiotics showed no antibacterial activity against these bacteria, except amikacin which had an acceptable effectiveness. However, the ethanol extracts of the studied plants revealed different antibacterial effects against *A. hydrophila* which showed antibiotic resistant. *T. vulgaris* extract had the strongest effect, whereas *O. europea* extract had the weakest activity. The water and ether petroleum extracts had no antibacterial activities.

**Conclusions:**

Ethanol extracts of the studied plants had different antibacterial effects against antibiotic-resistant *A. hydrophila*. *T. vulgaris* had the highest activity, *R. officinalis* had the second, and *M. communis* and *A. falcate* were in the third place, while the *O. europea* had the weakest antibacterial activity.

## 1. Background

*Aeromonas hydrophila* is among the most common bacteria in freshwater habitats throughout the world (1). It causes a variety of zoonotic diseases in both human and fish (2). *A. hydrophila* is associated with cellulitis, an infection that causes inflammation in the skin tissue (2, 3). *Aeromonas* species are most commonly causative agents of gastroenteritis (4), an inflammation of respiratory tract, and diarrhea accompanied with high temperature (5). Sepsis is a fatal complication of *Aeromonas* infections. Feasible virulence factors of *Aeromonas* species include fimbrial and afimbrial adhesion molecules, hemolysins, enterotoxins, lipases and proteases (4).

*A. hydrophila* is hard to remove, because it is resistant to chlorine and refrigeration or cold temperatures (6). In addition, it is very toxic to many organisms because of its structure. When it enters the body of fish, amphibians, or humans, it travels via the bloodstream to the first available organs. It produces cytotoxic enterotoxin (Act) which is one of the major virulence factors. Its toxin is produced and secreted by the cell from a type II secretion system. The toxin binds to high-affinity receptors and undergoes oligomerization to form a heptameric pore-forming complex which allows passage of small molecules in the plasma membrane, resulting in permeabilization of the cell, cell death, and eventually tissue destruction. *A. hydrophila* is also known as an opportunistic pathogenic bacterium that is they only infect immunocompromised hosts (7).

The volatile oils of black pepper, clove, glycine max (1) merr and perry, geranium, nutmeg, oregano, *Thymus vulgaris* (8), and acetone and hexane extracts of *Pterocarpus angolensis*, *Syzygium cordatum*, and *Zornia milneana* showed inhibitory activity against all *Aeromonas* isolates (9). Also Guava and Neem extracts showed higher antimicrobial activity against Gram-negative bacteria such as *A. hydrophila *(10).

Researchers try to develop new broad-spectrum antibiotics against bacteria, because the extended use of antibiotics has led to drug resistance. It directed the authors to make new medical plants which are a rich source of many compounds such as polyphenols. Polyphenols are a group of highly hydroxylated phenolic compounds which exist in the extractive fraction of several plant components. Polyphenols are proved to have bactericide activities against a huge number of pathogenic bacteria. Polyphenols in plants include flavanols, flavonols, flavanones, flavones, anthocyanins, proanthocyanidins (tannins), hydroxystilbenes, and aurones (11). Flavonoids are now the subject of medical research. They have been reported to possess many useful properties, including anti-inflammatory, oestrogenic, enzyme inhibition, and antimicrobial effects (12).

Therefore, from the flavonoid family the chosen plants were the leaves of *Olea europea* Linn from the *Oleaceae*, *Myrtus communis* Linn back to *Liliaceae*, *Thymus vulgaris* from the *Laminaceae*, and *Rosmarinuis officinalis* Linn (*Rosemary. L*) and the flowers of *Achillea falcata* Linn back to the *Asteraceae*.

*R. officinalis*, contains a variety of compounds including carnosol, carnosic acid, rosmanol, 7-methyl-epirosmanol, isorosmanol, rosmadial, caffeic acid, 1,8 cineol, camphor and α-pinene, which have in vitro antimicrobial and antioxidant activities. 1,8 cineol, α-pinene and camphor have been identified as the most active antimicrobial components (13).

The *O. europea* Linn from *Oleaceae*, is an evergreen long-lasting fruit tree, and is rooted in the Mediterranean region (14). Olive leaves are a good source of several antioxidants including oleuropeoside compounds such as oleuropein and verbascoside, and flavonoid compounds such as luteolin, luteolin-7-glucoside, apigenin-7-glucoside, diosmetin, diosmetin-7-glucoside, rutin and catechin, and simple phenolic compounds such as tyrosol, hydroxytyrosol, vanillin, vanillic acid and caffeic acid (13).

*T. vulgaris L*. (thyme), locally known as “Zaatar”, a member of the family *Lamiaceae*, is widely used in traditional medicine for its expectorant, antitussive, antibroncholitic, antispasmodic, anthelmintic, carminative and diuretic effects. The aromatic and medicinal properties of the genus Thymus have made it one of the most popular plants all over the world. Thymus species are commonly used as herbal tea, flavoring agents and medicinal plants. The published results reveal that major volatile constituents obtained from the aerial parts of the plant are geranial, linalool, γ-terpineol, carvacrol, thymol and trans-thujan-4-ol/terpinen-4-ol. Recent studies have shown that Thymus species have strong antibacterial, antifungal, antiviral, anti-parasitic, spasmolytic and antioxidant activities (15).

Indigenous uses of *Achillea* species are diuretic, emmenagog agents, wound healing, curing stomachache, diarrhea and antispasmodic, and are also used in cosmetics. Recent studies reported that *Achillea* species had constituents such as flavonoids (aglycones and glycosides), sesquiterpene lactones and essential oils, the major constituents are 1, 8-cineole, camphor, piperitone and ascaridole (16). *M. communis* grows throughout the Mediterranean area and has been used for medicine, food and spice since the ancient times. In folk medicine, the fruit of the plant is used to treat various infectious diseases, including diarrhea and dysentery, whereas the leaves are used as antiseptic and anti-inflammatory agents, like mouthwash, they are also used to treat candidiasis, heal wounds, and in the therapy of urinary tract diseases. The leaves contain tannins, flavonoids such as quercetin, catechin and myricetin derivatives and volatile oils (17).

## 2. Objectives

The current research aimed to investigate the anti-bacterial activity of the examined plant extracts prepared from different parts of *O. europea*, *M. communis*, *T. vulgaris*, *R. officinalis*, and *A. falcata* that grow in Syria against *Aeromonas hydrophila* that causes the most dangerous bacterial diseases in fish.

## 3. Materials and Methods

### 3.1. Collection of Plant Material

*O. europea*, *M. communis*, and *T. vulgaris *leaves, and *A. falcata* flowers were collected in the early morning hours from June to August 2010 in Damascus rural areas, while the *R. officinalis* leaves were purchased from Damascus market and the identity was confirmed by Damauscs University. The plants were washed with cold distilled water, and then dried under hot air at a temperature not exceeding 60°C in the shadow. The samples were crushed properly by metal mortar until a fine homogeneous powder was obtained, kept in paper bags under free humidity conditions (1).

### 3.2. Preparing Plant Extracts

 Plant extracts were prepared separately by a continuous extraction apparatus (Soxhlet apparatus), the method already described by Wang (6) for preparing plant extracts by organic solvents as follows: 50 g of plant powder was placed inside the thimble-holder of Soxhlet apparatus in addition to 500 mL of each organic solvent (rate 1:10 w: v). Three different polar solvents have been selected to extract the components of the plants, water, absolute ethanol, then light petroleum. Extraction continued for four hours, until the solvent that came out of the thimble became colorless. Then to concentrate the extracts, the ethanol and petroleum ether extracts were dried using rotary vacuum evaporator at a temperature not exceeding 40°C, while the aqueous extract was dried using freeze dryer. The thick layer of the bottom was stored in sterile bottles at 4°C for further experiments. All the extracts were filter-sterilized using 0.45 μm membrane filters (Whatman, UK) (1).

### 3.3. Sampling Method

Four hundred and fifty Samples from the intestines of the infected *Cyprinus carpio* fish with dermal ulceration received at the Central Laboratory of Veterinary from Althoura branch of fish of Raqqa province were investigated for the presence of *A. hydrophila*. The samples were preserved at 2 to 4°C and transported to the laboratory immediately. Glassware and culturing tools were sterilized. The samples were placed in sterile tubes, fitted with a strap closure, and the card number included name of the sample, place, and date of collection.

### 3.4. Method of Culturing Pathological Samples

Media cultures were Prepared, then each sample was placed in a tube containing 5 mL of nutrient broth and incubated for 24 hours at 37°C (1, 18, 19).

### 3.5. Identification Method of the Bacteria

The bacteria were identified morphologically and biochemically. The API20E system was also used to support the identification process. *A. hydrophila* were isolated after the following steps: Microscopic examination was conducted, then the bacteria were cultured on blood agar and trypticase soya agar (TSA) (HiMedia, India), according to Cipriano method (1) where the bacteria from the nutrient broth was cultured, then incubated for 20 to 24 hours at 35 to 37°C (20, 21).

#### 3.5.1. Analytical Profile Index Technique (API 20E)

This technique was performed according to the manufacturer’s instructions (Bio Merieux, France). Oxidase, catalase, and indole tests were also conducted. Twenty three differentials Bergey's Manual of Systematic Bacteriology were performed by API 20E system (Analytical Profile Index, manufactured by Bio Merieux, France) as follows:

- Investigate the effectiveness of enzymes: 2. nitrophenyl-β D-galactopyranoside, arginine dihydrolase, oxidase.
- Decarboxylation interactions of amino acids: L-lysine, L-omithine;
- Deaminase interaction of the amino acid: L-tryptophan;
- Fermentation reactions of the following sugars: D-glucose, D-mannitol, Inositol, D-sorbitol, L-rhamnose, D-sucrose, D-melibiose, amygdaline arabinose;
- Production reactions: indole, asitoen;
- Reactions produce gases: hydrogen sulphide, nitrogen, nitrogen oxide;
- Study the interactions of Recipes: gelatin diluted, citrate use, hydrolysis of urea (urease).

### 3.6. Antimicrobial Susceptibility Tests

The antimicrobial susceptibility testing was done on Muller-Hinton agar by disc diffusion method (Kirby-Bauer disk diffusion susceptibility test protocol). The A. hydrophila isolates (178 samples) were individually tested against 18 antibiotics. The results were determined using the disk diffusion method (Becton Dickinson, Microbiology Systems, MD, USA) as described in the National Committee For Clinical Laboratory Standards (NCCLS2000). The tested antibiotics and concentration ranges were as follows: amikacin (30 µg), ampicillin (10 µg), cephalexin (30 µg), cephalothin (30 µg), doxycycline (30 µg), cefadroxil (30 µg), ciprofloxacin (5 µg), clindamycin (2 µg), chloramphenicol (30 µg), erythromycin (15 µg), gentamicin (10 µg), norfloxacin (10 µg), oxytetracycline (30 µg), pefloxacin (5 µg), oxacillin (1 µg), enrofloxacin (5 µg), tetracycline (30 µg) and amoxicillin (25 µg). The resistance breakpoints were the same as the ones defined by the National Committee for Clinical Laboratory Standards (NCCLS, 2000) for Gram-negative bacteria (20).

### 3.7. Bacterial Growth Inhibition Test of Plant Extracts by the Disk Diffusion Method

Five microliters of plants extracts 66 mg/mL (the disk concentration was 0.33 mg/tablet) diluted in ethanol, water, and petroleum ether were impregnated into 6 mm sterile filter paper disks (22). All extracts were filter-sterilized using 0.45 μm membrane filters (Whatman Co. UK). Control disks were also prepared with absolute ethanol, water, and petroleum ether. After culturing bacteria on Mueller-Hinton agar, the disks were placed on the same plates and incubated for 17 hours at 37˚C. The diameters of the inhibition zones were measured in millimeters, and compared with those of the control and standard susceptibility disks.

## 4. Results

### 4.1. Identification of the Bacteria

Bacterial samples were identified using the tests mentioned in Table 1 and the results were compared to Cipriano and Illanchezian (2).

**Table 1.** Results of the Microbiology Laboratory Tests

Test	Test Result
**Microscopic examination**	Gram-negative bacilli, motile (2)
**Blood agar**	β-hemolysis
**Tryptone soya agar (TSA)**	yellow pigments

#### 4.1.2. The results of biochemical tests 

The results of the biochemical tests were oxidase positive (2), catalase positive (21), and indol positive (17). The results of Analytical Profile Index technique (API20E) are displayed in Table 2.

**Table 2.** Results of the Biochemical Tests based on (API20E) Technique

Test	Test Result
**Citrate**	+
**lysine decarboxylase**	+
**Ornithine decarboxylase**	-
**Urease**	-
**Phenylalanine (TDA)**	-
**Nitrate production**	+
**Hydrogen sulphide (H**_**2**_ **S) production**	+
**Glucose fermentation**	+
**Lactose fermentation**	-
**Arabinose fermentation**	+
**sorbitol fermentation**	-

### 4.2. Antibiotic Susceptibility Results Against A. hydrophila

*A. hydrophila,* a species from genus *Aeromonase*, were found resistant to the studied antibiotics, except for amikacin. The diameter of inhibition zones were measured, based on NCCLS 2000 criteria (20), and the standard leaflet of antibiotic discs provided by the manufacturer and the result was 23 ± 1.2 mm. The number of infected samples and the rate of sensitive and resistant isolates of *A. hydrophila* (178 samples) against the examined antibiotics is shown in Tables 3, 4, respectively.

**Table 3.** Number of Infected Samples

	No. of Infected Samples (%)	
**samples infected with ** ***Aeromonas*** ** spp **	330 (73.33)	
**samples infected with ** ***A.hydrophila***	178 (39.55)	

**Table 4.** Growth Inhibition Zone of the Examined Antibiotics^a^

Antibiotics	Diameters Zones of Inhibition, mm	Antimicrobial Susceptibility Results	Sensitive Bacteria, %
**T**	6-8	resistant	98
**AX**	6-9	resistant	99
**OX**	7-10	resistant	97
**CER**	6-8	resistant	99
**PEF**	7-9	resistant	96.7
**AK**	21-24	sensitive	98
**TE**	5-7	resistant	97.11
**CIP**	7-10	resistant	89.77
**NOR**	5-8	resistant	95.98
**CN**	6-8	resistant	94.55
**C**	6-8	resistant	97.45
**ENR**	7-9	resistant	96.87
**DO**	6-9	resistant	97.08
**CL**	6-9	resistant	94.45
**KF**	5-7	resistant	95.86
**DA**	7-9	resistant	88.59
**AM**	6-8	resistant	91.61
**E**	5-9	resistant	98.75

^a^ Abbreviations: AM, ampicillin; AK, amikacin; AX, amoxicillin; C, chloramphenicol; CER, cefadroxil; CIP, ciprofloxacin; CL, cephalexin; CN, gentamycin; DA, clindamycin; DO, doxycycline; E, erythromycin; ENR, enrofloxacin; KF, cephalothin; NOR, norfloxacin; OX, oxacillin; PEF, pefloxacin; T, oxytetracycline; TE, tetracycline

### 4.3. Antibacterial Activity of Plant Extracts Against A. hydrophila. 

The ethanol extracts showed the antibacterial activity, on the contrary, the water and ether petroleum extracts had no antibacterial effects (no inhibition zone was seen). Table 5 is shown the results obtained from the antibacterial effects of the various examined extracts, in comparison with those of the control disks, through measuring the diameter of inhibition zone of antibiotic-resistant *A. hydrophila* isolates. 

**Table 5.** Antibacterial Activity of Examined Plant extracts Against *A. hydrophila*

Plant	Inhibition Zones of Plant Ethanol Extracts, mm	Sensitive Bacteria, %
**Control/ 5 µm**	0	0
***Olea europea *** **leaves**	10-13	69.12
***Myrtus communis *** **leaves**	13-15	96.89
***Thymus vulgaris *** **leaves**	30-33	97.76
***Rosemery*** ** leaves**	24-27	98
***Achillea falcata*** ** flowers**	12-15	97.08

## 5. Discussion

In the current study, *Aeromonas* isolates were observed in 330 (73.33%) of the infected fishes. Out of 450 fish samples, 178 (39.55%) *A. hydrophila* isolates were obtained that is. similar to Citarasu et al. study with 80% *Aeromonas *spp., and 35% *A. hydrophila* (23). Recently, the development of multi-resistant bacteria against different antibiotics has increased. As observed in the current study (Table 4), there was resistance against all antibiotics, except amikacin, whose inhibition zone was 21 to 24 mm. The obtained results comply with those of Andrea Belem-Costa et al. study, who showed resistance of *A. hydrophila* against amoxicillin, ampicillin, and oxacillin (24). However, other studies have shown the resistance of *A. hydrophila* to enrofloxacin and/or ciprofloxacin (25), and also amikacin, tobramycin, and cephalosporins (26), which were not observed in the current study. 

Resistance to antibiotics led the authors to develop alternative antimicrobial medicines to treat infectious diseases. So they used different Plant extracts derived from five plants. In the current study, the ethanol extracts of the five plants exhibited disparate antibacterial activities, while no effect for water, and petroleum ether extracts was observed. The diameter of inhibition zone was 30 to 33 mm. Fabio`s study showed the greater antibacterial activity of thyme volatile oil than the ethanol extract of thyme leaves against *A. hydrophila*. The growth inhibition zone of the ethanol extract of rosemary was 24 to 27 mm, whereas the volatile oil of rosemary had no effect on *A. hydrophila *(27). Moreno et al. reported that rosemary is a rich source for phenolic compounds with high antimicrobial activity against both Gram-positive and Gram-negative bacteria. They attributed the high antimicrobial activity of carnosic acid and carnosol. Carnosic acid and rosmarinic acid may be the main bioactive antimicrobial compounds which exist in rosemary extracts (28).

Essential oils derived from thymus are found to possess significant antifungal, insecticidal, and antimicrobial effects. These properties greatly depend on the chemicals attributed to their contents at phenolic compounds (thymol and carvacrol) (29). P-cymene (precursor of carvacrol) swells bacterial cell membranes to a greater extent than carvacrol does. By this mechanism p-cymene probably enables carvacrol to be more easily transported into the cell, so that a synergistic effect is achieved when the both are used together (30).

*M. communis*, and *A.falcata* had the same impact, 13 to15 mm growth inhibition zone diameters. Authors believe that this is the first study on the antibacterial effect of these Plant extracts on *A. hydrophila*. It is often reported that Gram-negative bacteria are more resistant to the Plant -based extracts and essential oils (31), since the hydrophilic cell wall structure of Gram-negative bacteria is constituted essentially of a lipo-polysaccharide (LPS) that blocks the penetration of hydrophobic oil and avoids the accumulation of essential oils in the target cell membrane (32). This is the reason that Gram-positive bacteria were found to be more sensitive to the extracts of *M. communis* than Gram-negative ones (33) and the essential oil of *A. falcata* shows inhibitory effects mainly against Gram-positive bacteria (34). *M. communis* leaves contain different polyphenolic classes, as flavonols and galloyl derivatives, and their antimicrobial mode of action is related to the presence of phenolic compounds. Most of the studies on the mechanism of phenolic compounds have focused on their effects on cellular membranes. They have been observed not only attacking cell walls and cell membranes, affecting their permeability and releasing intracellular constituents, but also interfering with membrane functions such as electron transport, and enzyme effect or nutrient uptake. Thus, active phenolic compounds might have several targets which could lead to the inhibition of bacteria growth (33). Moreover, *M. comlone* A&B are considered as two new acylphloraglucinols identified in the leaves and fruits of *M. communis* and have shown a significant antimicrobial effect against bacteria. The inhibitory effect may be due to presence of tannin through producing hydrogen bonds with proteins, which converted its structure and led to block the protein synthesis, and tannins were considered as a phenolic compounds of plants with anti-oxidative effects (35).

Alishahi et al. revealed that the ethanol extract of *O. europea* has a little effect against this type of bacteria (10 to 12 mm) (36) which confirmed the results of the current study. Oleuropein has shown to have strong antimicrobial activity against both Gram-negative and Gram-positive bacteria, as well as Mycoplasma species. Phenolic structures similar to those of oleuropein seem to have antibacterial activities through damaging the bacterial membrane and/or disrupting cell peptidoglycans (37). However, the exact mechanism of the antimicrobial effect of oleuropein is not completely confirmed yet, although some authors have proposed that it is due to the presence of the ortho-diphenolic system (catechol). Omar SH proposed that the glycoside group changes the ability to penetrate the cell membrane and get at the target site. Effective interference with the production procedures of certain amino acids necessary for the growth of specific microorganisms has been also suggested. Another proposed mechanism is the direct stimulation of phagocytosis as a response of the immune system to microbes of all types (38). Finally, plants were arranged according to their activity against *A. hydrophila* and it was found that thyme had the highest effect, rosemary had the second place, myrtus and Achilles were in the third while the olive was in the last place and had the lowest activity. Thyme showed more antibacterial activity against the examined bacteria than the strongest antibiotic amikacin; in a way that bacterial growth zone was 30 to 33 mm, and was 21 to 24 mm for the thyme and amikacin, respectively.

## 6. Conclusions

The examined plant ethanol extracts showed different antibacterial activities against antibiotic-resistant *A. hydrophila*, while plant water and ether petroleum extracts had no antibacterial activities.
